# METTL14 regulates CD8^+^T-cell activation and immune responses to anti‐PD‐1 therapy in lung cancer

**DOI:** 10.1186/s12957-024-03402-9

**Published:** 2024-05-10

**Authors:** Chongqi Sun, Jian Wang, Huixing Li, Luyao Liu, Yu Lin, Ling Zhang, Xianglong Zu, Yizhi Zhu, Yongqian Shu, Dong Shen, Qiong Wang, Yiqian Liu

**Affiliations:** 1 Department of Oncology, The First Affiliated Hospital of Nanjing Medical University, Nanjing, Jiangsu 210029 China; 2 Department of Oncology, Wuxi Second Geriatric Hospital, Wuxi, Jiangsu 214174 China; 3 Department of Thoracic Surgery, The Affiliated Wuxi People’s Hospital of Nanjing Medical University, Wuxi, Jiangsu 214023 China; 4 Department of Oncology, The Affiliated Suzhou Hospital of Nanjing Medical University, Suzhou, Jiangsu 215001 China; 5 Department of Oncology, Jintan Hospital Affiliated to Jiangsu University, Changzhou, Jiangsu 213200 China; 6 Nanjing Medical University, Nanjing, Jiangsu 210029 China; 7 Department of Oncology, The Affiliated Jiangyin Hospital of Nantong University, Jiangyin, Jiangsu 214400 China

**Keywords:** METTL14, Lung cancer, PD-1, CD8^+^ T cell, HSD17B6

## Abstract

**Background:**

N6-methyladenosine (m6A) modification plays an important role in lung cancer. However, methyltransferase-like 14 (METTL14), which serves as the main component of the m6A complex, has been less reported to be involved in the immune microenvironment of lung cancer. This study aimed to analyze the relationship between METTL14 and the immune checkpoint inhibitor programmed death receptor 1 (PD-1) in lung cancer.

**Methods:**

CCK-8, colony formation, transwell, wound healing, and flow cytometry assays were performed to explore the role of METTL14 in lung cancer progression in vitro. Furthermore, syngeneic model mice were treated with sh-METTL14 andan anti-PD-1 antibody to observe the effect of METTL14 on immunotherapy. Flow cytometry and immunohistochemical (IHC) staining were used to detect CD8 expression. RIP and MeRIP were performed to assess the relationship between METTL14 and HSD17B6. LLC cells and activated mouse PBMCs were cocultured in vitro to mimic immune cell infiltration in the tumor microenvironment. ELISA was used to detect IFN-γ and TNF-α levels.

**Results:**

The online database GEPIA showed that high METTL14 expression indicated a poor prognosis in patients with lung cancer. In vitro assays suggested that METTL14 knockdown suppressed lung cancer progression. In vivo assays revealed that METTL14 knockdown inhibited tumor growth and enhanced the response to PD-1 immunotherapy. Furthermore, METTL14 knockdown enhanced CD8^+^T-cell activation and infiltration. More importantly, METTL14 knockdown increased the stability of HSD17B6 mRNA by reducing its m6A methylation. In addition, HSD17B6 overexpression promoted the activation of CD8^+^ T cells.

**Conclusion:**

The disruption of METTL14 contributed to CD8^+^T-cell activation and the immunotherapy response to PD-1 via m6A modification of HSD17B6, thereby suppressing lung cancer progression.

**Supplementary Information:**

The online version contains supplementary material available at 10.1186/s12957-024-03402-9.

## Introduction

Lung cancer is one of the most common malignant tumors worldwide [1]. GLOBOCAN estimated that there were approximately 2.2 million new cases of lung cancer in 2020, accounting for 11.4% of all malignancies, and approximately 1.8 million deaths, accounting for 18.0% of cancer-related deaths [2]. Many lung cancer patients are diagnosed as advanced stage with metastases and have lost the opportunity for surgical treatment [3, 4]. At present, the clinical treatment methods for early lung cancer mainly include surgery, radiotherapy, chemotherapy, molecular targeted therapy and immunotherapy [3, 4]. Despite the development of multiple treatments, the 5-year survival rate for patients with lung cancer remains low. Therefore, it is urgent to find effective methods to treat lung cancer.

Immunotherapy involves the reconstruction of the tumor immune system, which depends on the interaction between tumor cells and infiltrating immune cells and on the degree of infiltration of immune cells in the tumor microenvironment [5]. The tumor microenvironment consists of cancer cells and stromal cells, including fibroblasts, endothelial cells and immune cells, which provide a sustained environment for cancer cell proliferation [6]. In recent years, great progress has been made in the treatment of lung cancer via tumor immunotherapy, mainly including the use of genetically engineered T cells and immune checkpoint blockers [7]. The T-cell-mediated cellular immune response plays an important role in tumor immunity [8]. T-cell dysfunction and immune escape are the main causes of lung cancer [9]. Studies have shown that CD8^+^ T cells mainly play a role in killing tumor cells in the tumor microenvironment [10]. Therefore, the in-depth study of the functional status of CD8^+^ T cells in the tumor microenvironment provides hope for the immunotherapy of lung cancer.

The immune checkpoint is an important immune regulator that maintains immune homeostasis and prevents autoimmune diseases [11]. Programmed death receptor 1 (PD-1) is a relatively mature immune checkpoint molecule that belongs to the immunoglobulin superfamily and is mainly expressed on the surface of T cells [12, 13]. Programmed death-ligand 1 (PD-L1) is the receptor for PD-1 and is mainly expressed on the surface of antigen-presenting cells or tumor cells [12, 13]. Immunotherapy, represented by immune checkpoint inhibitors such as PD-1 and PD-L1, has been shown to improve survival in lung cancer patients [12]. The extracellular interaction between PD-1 and PD-L1 inhibits the T-cell killing response and leads to immune escape of tumor cells [14].

N6-methyladenosine (m6A) is the most abundant type of RNA modification and plays an important role in RNA metabolism and various biological processes in cells [15]. M6A modification is a dynamic and reversible posttranscriptional modification process mediated by a group of proteins, including methyltransferase "writers" (WTAP, METTL3 and METTL14), demethylase "erasers" (FTO and ALKBH5) and methyl-recognition protein "readers" (YTHDF1, YTHDF2, YTHDF3, YTHDC1 and YTHDC2) [15]. Studies have confirmed that abnormal m6A modification may be a potential mechanism underlying the occurrence and development of several tumors [16]. Methyltransferase-like 14 (METTL14) is a central component of the m6A methyltransferase complex and is abnormally expressed in a variety of tumors [17]. Furthermore, dysregulation of METTL14 plays an important role in the progression of various cancers [17]. A previous study reported that METTL14-mediated m6A modification facilitated NSCLC cell resistance to cisplatin via the miR-19a-5p/RBM24/AXIN1 axis [18]. Macrophage-specific knockout of METTL14 promoted CD8^+^T-cell differentiation along a dysfunctional trajectory, impairing the ability of CD8^+^ T cells to eliminate tumors [19]. However, there are few studies on the effects of METTL14 on the lung cancer immune microenvironment.

Therefore, the present study aimed to analyze the relationship between the m6A methyltransferase METTL14 and PD-1 in lung cancer. Here, the disruption of METTL14 contributed to CD8^+^T-cell activation and the immunotherapy response to PD-1, thereby suppressing lung cancer progression.

## Materials and methods

### Cell culture

A mouse Lewis lung carcinoma (LLC) cell line and mouse peripheral blood mononuclear cells (PBMCs) were purchased from the Cell Bank of the Chinese Academy of Sciences (Shanghai, China). LLC cells were maintained in Dulbecco’s modified Eagle’s medium (DMEM, Gibco, USA) supplemented with 10% fetal bovine serum (FBS, Gibco, USA) at 37 °C under 5% CO_2_.

PBMCs were cultured in RPMI-1640 medium (Gibco, USA) supplemented with 10% FBS (Gibco, USA) at 37 °C under 5% CO_2_. A MagniSort™ Mouse CD8^+^T-cell Enrichment Kit (Invitrogen, USA) was used for negative selection of mouse CD8^+^ T cells. Then, CD8^+^ T cells were activated with Dynabeads™ Mouse T-Activator CD3/CD28 (Gibco, USA). CD8^+^ T cells and LLC cells were cocultured for 48 h. In brief, activated CD8^+^ T cells were resuspended in serum-free RPMI 1640 medium in the upper chamber of a transwell plate (5 mm), and LLC cells were cultured in the lower chamber of a transwell chamber.

### Cell transfection

For the knockdown of METTL14, YTHDF1, YTHDF2 and YTHDF3, short hairpin RNAs (shRNAs) against METTL14, YTHDF1, YTHDF2, and YTHDF3 (sh-METTL14, sh-YTHDF1, sh-YTHDF2, and sh-YTHDF3) and a negative control (sh-NC) were purchased from RiboBio (Guangzhou, China). For overexpression of METTL14 and HSD17B6, the cDNA sequences of METTL14 and HSD17B6 were cloned and inserted into the pLV-EF1a-EGFP(2A) Puro vector by RiboBio (Guangzhou, China). LLC cells were seeded at 1 × 10^5^ cells/well in a 24-well plate. When the percentage of LLC cells was approximately 70%, the cells were transfected with 1 μg of shRNA or 50 pmol of plasmid for 24 h or 48 h using Lipofectamine 2000 (Invitrogen, USA) following the manufacturer's instructions.

### CCK-8

LLC cells were seeded in 96-well plates at a density of 2 × 10^3^ cells/well. LLC cells were incubated with 100 μL of CCK8 reagent (Abcam, USA) for 4 h at 24 h, 48 h, 72 h, and 96 h. The optical density was measured at 450 nm using a microplate reader (Bio-Rad, USA).

### Clone formation

LLC cells were seeded at 5 × 10^3^ cells/well in 6-well plates and cultured at 37 °C for 2–3 weeks. The cell culture was terminated when the clone was visible to the naked eye. The supernatant was removed, and the cells were washed twice with PBS. The cells were fixed with 4% paraformaldehyde for 15 min and stained with 0.1% crystal violet staining solution for 10–30 min. Ten random fields of view were observed under a light microscope (Olympus, Japan), and the number of colonies was counted.

### Transwell

For cell migration, LLC cells were cultured in serum-free medium in a transwell upper chamber with an 8 μm pore size, and DMEM medium containing 20% FBS was added to the transwell lower chamber. For cell invasion, LLC cells were cultured in serum-free medium in a transwell upper chamber pretreated with Matrigel (BD Biosciences, USA), and DMEM medium containing 20% FBS was added to the lower chamber. The cells were fixed with 4% paraformaldehyde for 15 min and stained with 0.1% crystal violet for 15 min. The migrated and invasive cells were counted under a microscope (Olympus, Japan) and analyzed statistically.

### Wound healing

LLC cells were seeded into 24-well plates at a density of 2 × 10^5^ cells/well and incubated at 37°Cfor 12 h. The wound location was recorded by a sterile tip. After 24 h, the marked wound location was photographed by a microscope (Olympus, Japan) to assess the cell migration ability.

### Flow cytometry for cell apoptosis

A total of 1 × 10^5^ cells were centrifuged at 1200 rpm for 5 min to remove the supernatant. Then, 50 μL of binding buffer (Beyotime, China) was added to each sample tube, and the sample was resuspended. Then, 5 μL of Annexin V-FITC (Beyotime, China) and 5 μL of PI (Beyotime, China) were added to the sample tube. The solution was gently shaken and incubated for 15 min at room temperature in the dark. Then, 200 μL of 1 × binding buffer (Beyotime, China) was added, and cell apoptosis was detected by a FACSCalibur flow cytometer (BD Biosciences, USA).

### RNA immunoprecipitation (RIP) assay

The RIP assay was carried out with a Magna RIP Kit (Millipore, USA). LLC cells were lysed with RIP lysate for 5 min on ice, and then the cell lysates were centrifuged at 1500 rpm for 5 min. The supernatant was collected and precleaned with protein A-Sepharose beads. RIP wash buffer was used to wash the magnetic beads. RIP lysates were immunoprecipitated with 5 μg of METTL14, YTHDF2 or IgG antibody (negative control). Then, 900 μL of RIP immunoprecipitation buffer was added to the magnetic beads. Then, 100 μL of the supernatant was added to the bead-to-antibody complex in the previous step for a total volume of 1 mL. The above magnetic bead-antibody complex was resuspended in 150 μL of proteinase K buffer at 55 °C for 30 min. Then, 250 μL of RIP wash buffer was added to the supernatant. Finally, RNA was extracted via the phenol‒chloroform method. Agarose gel electrophoresis was used to detect HSD17B6 expression.

### Immunoprecipitation of m6A-methylated RNA (MeRIP)

MeRIP was measured by the MeRIP™ m6A Transcriptome Profiling Kit (RiboBio, China) according to the manufacturer’s instructions. Briefly, 18 μg of total RNA was added to 2 μL of 10 × RNA fragmentation buffer, and the reaction was performed at 70 °C for 7 min. The segmented RNA was added to 2 μL of 0.5 M EDTA. The reaction products were added to 18 μL of 3 M sodium acetate (pH 5.2),1 μL of glycogen (20 mg/mL) and 600 μL of absolute ethanol and precipitated overnight at -20 °C. The precipitated product was centrifuged at 12,000 × g at 4 °C for 30 min and washed twice with 75% ethanol. RNA was dissolved in 50 μL of nuclease-free water. Then, 500 μL of the MeRIP reaction solution was added to the prepared anti-m6A magnetic beads and incubated for 2 h. After the IP, 100 μL of the eluent buffer was added to the sample, which was gently blown and mixed to fully resuspend it. The eluted RNA was recovered, and the IP group RNA was captured by a m6A antibody. After immunoprecipitation, a portion of the RNA was analyzed via qRT‒PCR.

### RNA stability

To detect RNA stability in cells, LLC cells were transfected with METTL14 overexpression lentivirus and sh-YTHDF2 for 24 h, and then 5 mg/mL actinomycin D (Sigma, MA, USA) was added to the cells. RNA was isolated using TRIzol reagent (Invitrogen, NY, USA) at 0, 2, 4, and 6 h, analyzed by real-time PCR, and normalized to GAPDH. The t1/2 of HSD17B6 was calculated.

### Enzyme-linked immunosorbent assay (ELISA)

The levels of IFN-γ and TNF-α were measured using an IFN-γ ELISA Kit (Beyotime, China) and a TNF-α ELISA Kit (Beyotime, China), respectively,according to the manufacturer’s instructions.

### Experimental animals

The animal experiments were approved by the Animal Care and Use Committee ofNanjing Medical University (IACUC-1706007). Eighty male BALB/c mice (SPF grade 18–22 g) were purchased from Beijing Weitong Lihua Laboratory Animal Technology (Beijing, China). Male BALB/c mice were housed with fed ordinary diet and sufficient drinking water at 18–22 °C and a relative humidity of 40%-70%.

After one week of acclimation, the subcutaneous tumor model was constructed, and a total of 30 male BALB/c mice were divided into 6 groups: 1). sh-Ctrl, sh-METTL14; 2) sh-Ctrl, sh-Ctrl + anti-PD-1, sh-METTL14, sh-METTL14 + anti-PD-1. The other 20 mice were divided into sh-Ctrl and sh-METTL14 groups, and the survival conditions within 40 days of modeling were recorded.

LLC cells were transfected and prepared into 1 × 10^6^ cells/ml single-cell suspensions in PBS after the corresponding transfection treatments. LLC cells were inoculated subcutaneously into BALB/c mice in a sterile environment, and the LLC lung cancer mouse model was successfully established after nodules with a diameter greater than 5 mm^3^ appeared subcutaneously in the vaccinated mice. For mice in the PD-1 antibody treatment group, 10 mg/kg anti-PD-1 (Abcam, USA) was injected intraperitoneally on days 11, 14, 17, 20 and 23. An equal amount of IgG was injected as a control.

### Immunohistochemical (IHC) staining

The removed tumor tissues were fixed in 4% paraformaldehyde for 24 h. Then, the tissues were embedded in paraffin and sectioned. The paraffin-embedded tumor sections were dewaxed and treated with 3% H_2_O_2_ to deactivate endogenous peroxidase. After blocking with goat serum for 1 h, the sections were incubated with an anti-CD8antibody (1:500, Abcam, USA) at 4 °C overnight. After the sections were incubated with goat anti-mouse IgG H&L (HRP) at 37 °C for 30 min, they were stained with DAB and counterstained with hematoxylin. Images of stained sections were observed under a light microscope (Nikon, Japan).

### Flow cytometry for cell composition

After the mice were sacrificed, the tumor tissues were collected,minced into small pieces of 1 mm^3^ and placed in Petri dishes. Collagenase (Sigma, USA) was added to the tissue, which was subsequently digested at 37 °C for 20 min to isolate the cells. The isolated cells were incubated with Alexa Fluor® 647 Fluorescent Anti-CD8 (1:1000, Abcam, USA), Alexa Fluor®488 Fluorescent Anti-CD4 (1:1000, Abcam, USA), and FITC Fluorescent Anti-CD45 (1:1000, Abcam, USA) at4°C in the dark for 30 min. The cell precipitate was resuspended in 400 μL of PBS and detected by a FACSCalibur Flow Cytometer (BD Biosciences, USA).

### RT‒qPCR

Total RNA was extracted from cells and tissues using TRIzol reagent (TIANGEN, China). Then, the RNA was reverse-transcribed to cDNA using a PrimeScript™ RT kit (Takara, Japan). cDNA (50 pg) was amplified by an ABI Real-Time PCR System (Applied Biosystems, USA) using SYBR Premix Ex TaqII (Takara, USA). The results were calculated using the 2^−ΔΔCt^ method, and GAPDH was used as a control. The sequences of the primers used are shown in Table 1.

**Table 1:** Primer sequences

Gene	Forward primer (5′-3′)	Reverse primer (5′-3′)
MYH11	ATGAGGTGGTCGTGGAGTTG	GCCTGAGAAGTATCGCTCCC
LRRK2	AGCCTTGGATCTCCTCCTAGA	ACGTACTCAGCAGTATCGTGTAA
HSD17B6	TGGGGTTTGGTTAATAATGCAGG	GATAGGCATGTAGTCCTCTGGT
A2M	GGCAGAATTTCCGCTTAGAGG	CACACACGGACACATTCATCT
Cxcl15	TCGAGACCATTTACTGCAACAG	CATTGCCGGTGGAAATTCCTT
GAPDH	GCACCGTCAAGGCTGAGAAC	TGGTGAAGAACGCCAGTGGA

### Western blot

Total protein was extracted from cells and tissues using RIPA buffer (Beyotime, China). Then, the protein concentration was detected by a BCA assay kit (Santa Cruz, USA). Protein samples (40 μg) were separated by SDS‒PAGE and then transferred to PVDF membranes. After blocking, the membranes were incubated with primary antibodies overnight at 4 °C and with a goat anti-mouse IgG H&L (HRP)-preadsorbed secondary antibody for 1 h at room temperature. Finally, enhanced chemiluminescence (ECL, Thermo Fisher, MA, USA) was used to visualize the membrane. Protein band analysis was conducted with ImageJ software. The following antibodies were used: anti-Granzyme B (1:1000, ab283315, Abcam, USA); anti-METTL14 (1:1000, ab220030, Abcam, USA); anti-HSD17B6 (1:1000, orb539880, Biorbyt, USA); anti-Perforin (1:1000, ab47225, Abcam, USA); anti-GAPDH (1:2000, ab8245, Abcam, USA); and goat anti-mouse IgG H&L (HRP)-preadsorbed secondary antibody (1:5000, ab47827, Abcam, USA).

### Statistical analysis

All experimental data are displayed as the mean ± standard deviation (SD) of 3 independent experiments. Student’s ttest was used to compare the differences between two groups, and one-way ANOVA followed by Tukey’s post hoc test was used to analyze differences among multiple groups. *P* < 0.05 was considered to indicate statistical significance.

## Results

### High METTL14 expression indicates poor prognosis in lung cancer patients

**Fig. 1:**
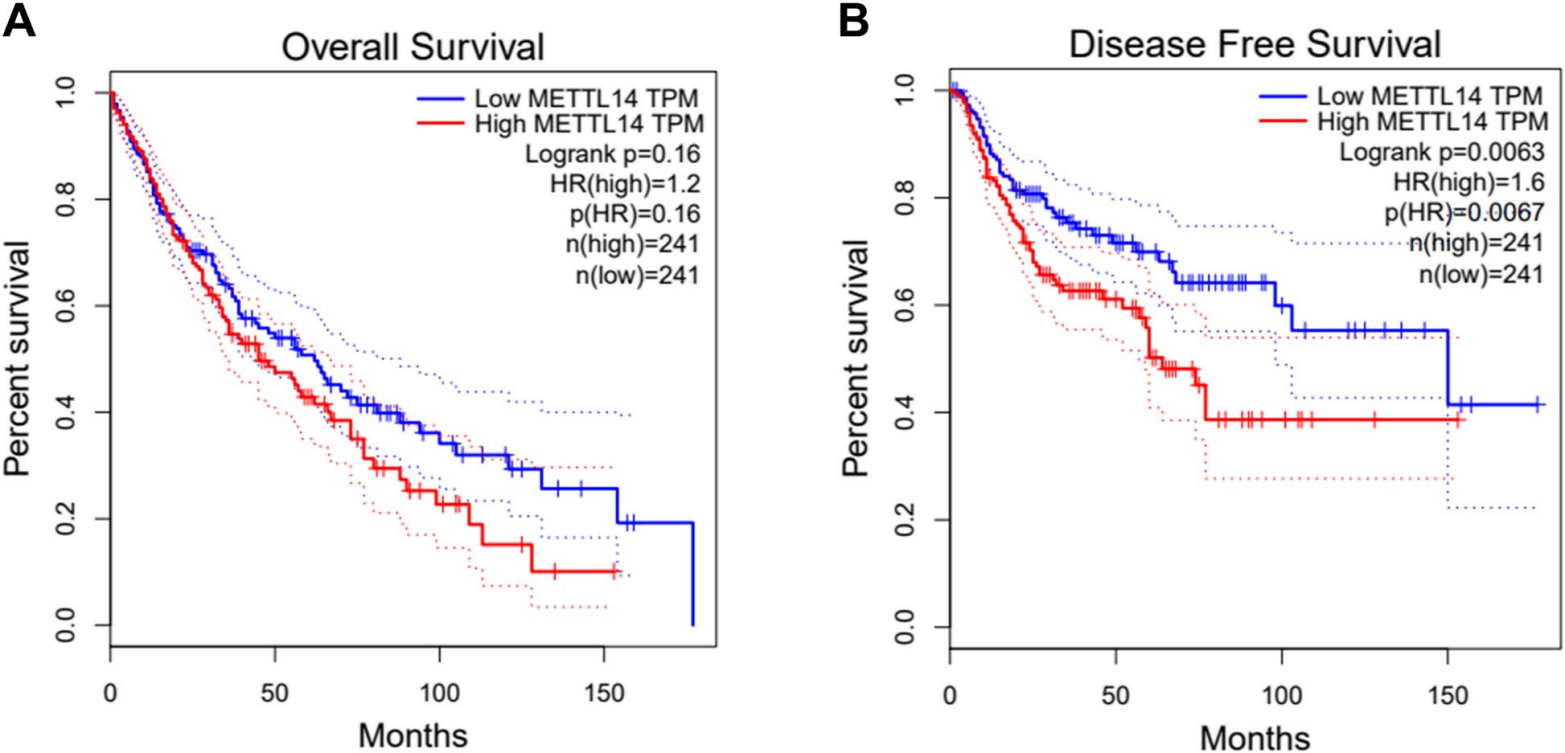
High METTL14 expression indicates poor prognosis in lung cancer patients. **A** The online database GEPIA was used to predict overall survival (OS) in lung cancer patients with high METTL14 expression and low METTL14 expression. **B** The online database GEPIA was used to predict disease-free survival (DFS) in lung cancer patients with high METTL14 expression and low METTL14 expression

The online database GEPIA (http://gepia.cancer-pku.cn/index.html) showed that lung cancer patients with high METTL14 expression had shorter overall survival (OS) than patients with low METTL14 expression (Fig. 1A). In addition, the disease-free survival (DFS) of lung cancer patients with high METTL14 expression was lower than that of patients with low METTL14 expression (Fig. 1B). These online prediction results indicated that the level of METTL14 was closely related to the survival of lung cancer patients.

### METTL14 knockdown inhibits the carcinogenicity of LLC cells

**Fig. 2:**
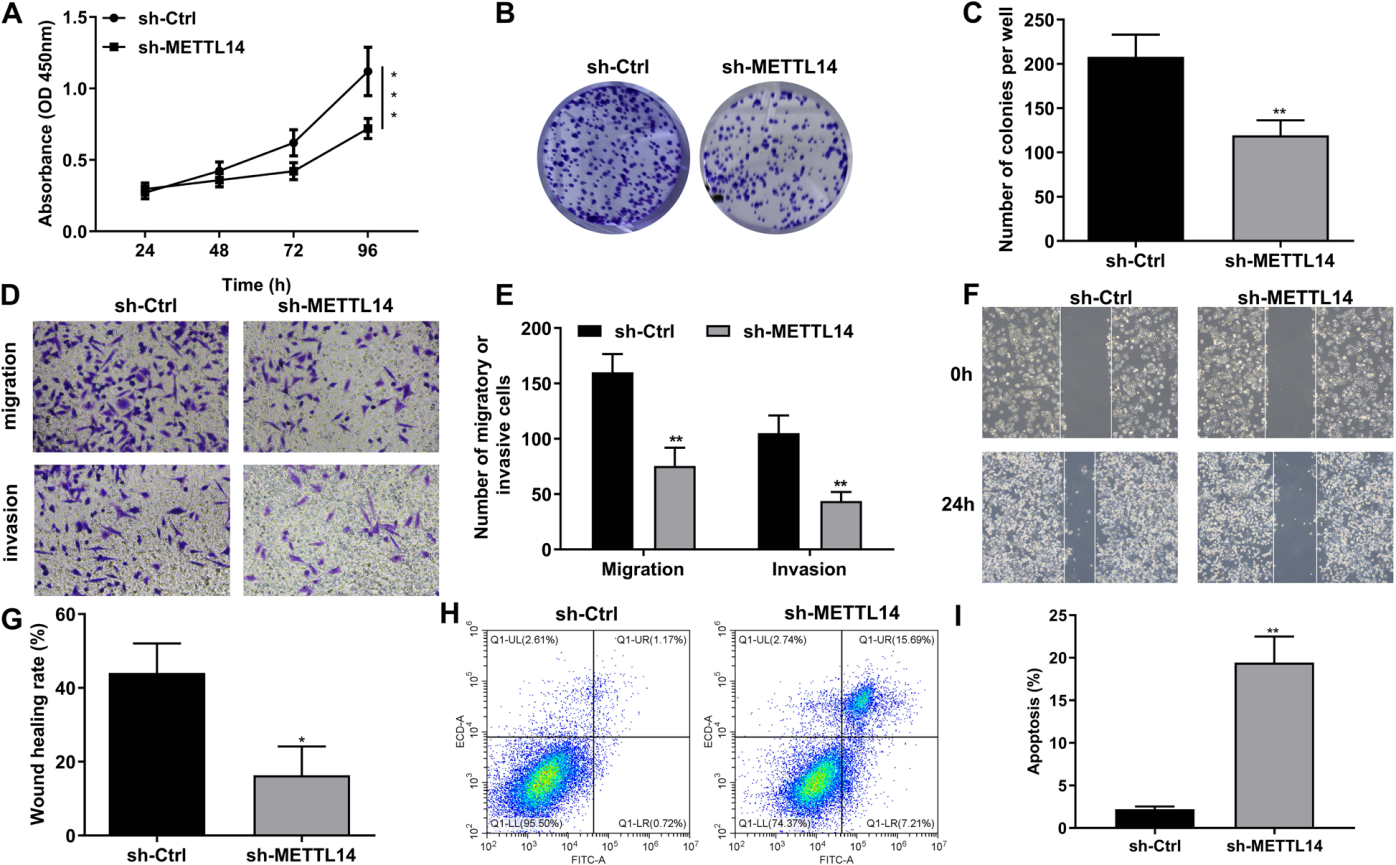
Knockdown of METTL14 inhibits the carcinogenicity of LLC cells. LLC cells were transfected with sh-METTL14 for 24 h. **A **The viability of LLC cells with METTL14 knockdown was assessed via a CCK-8 assay. **B** The proliferation ability of LLC cells with METTL14 knockdown was detected by clone formation assays. **C** Quantification of clone formation. **D** Transwell assays were performed to assess the migration and invasion of LLC cells with METTL14 knockdown. **E** Quantification of the transwell assay results. **F** Wound healing assays were carried out to detect LLC cell migration ability. **G** Quantification of wound healing. **H** Flow cytometry was performed to measure the apoptosis rate of LLC cells with METTL14 knockdown. **I** Quantification of flow cytometry data. ^*^P < 0.05, ^**^P < 0.01, ^***^P < 0.001 vs. sh-Ctrl

To explore the role of METTL14 in lung cancer progression, LLC cells were transfected with sh-METTL14 for 24 h, and changes in LLC cell function were studied. CCK-8 assays showed that METTL14 knockdown significantly decreased LLC cell viability (*P* < 0.001, Fig. 2A). In addition, clone formation assay suggested that METTL14 knockdown reduced the proliferation of LLC cells (*P* < 0.01, Fig. 2A-B). Transwell assays verified the decreased invasion and migration of LLC cells treated with sh-METTL14 (*P* < 0.01, Fig. 2D-E). Moreover, a wound healing assay verified that LLC cell migration decreased after METTL14 knockdown (*P* < 0.05, Fig. 2F-G). The LLC cell apoptosis rate also significantly increased after METTL14 knockdown (*P* < 0.01, Fig. 2H-I). These results consistently indicated that METTL14 suppression significantly inhibited lung cancer progression in vitro.

### METTL14 knockdown inhibits tumor growth and enhances the response to PD-1 immunotherapy

**Fig. 3:**
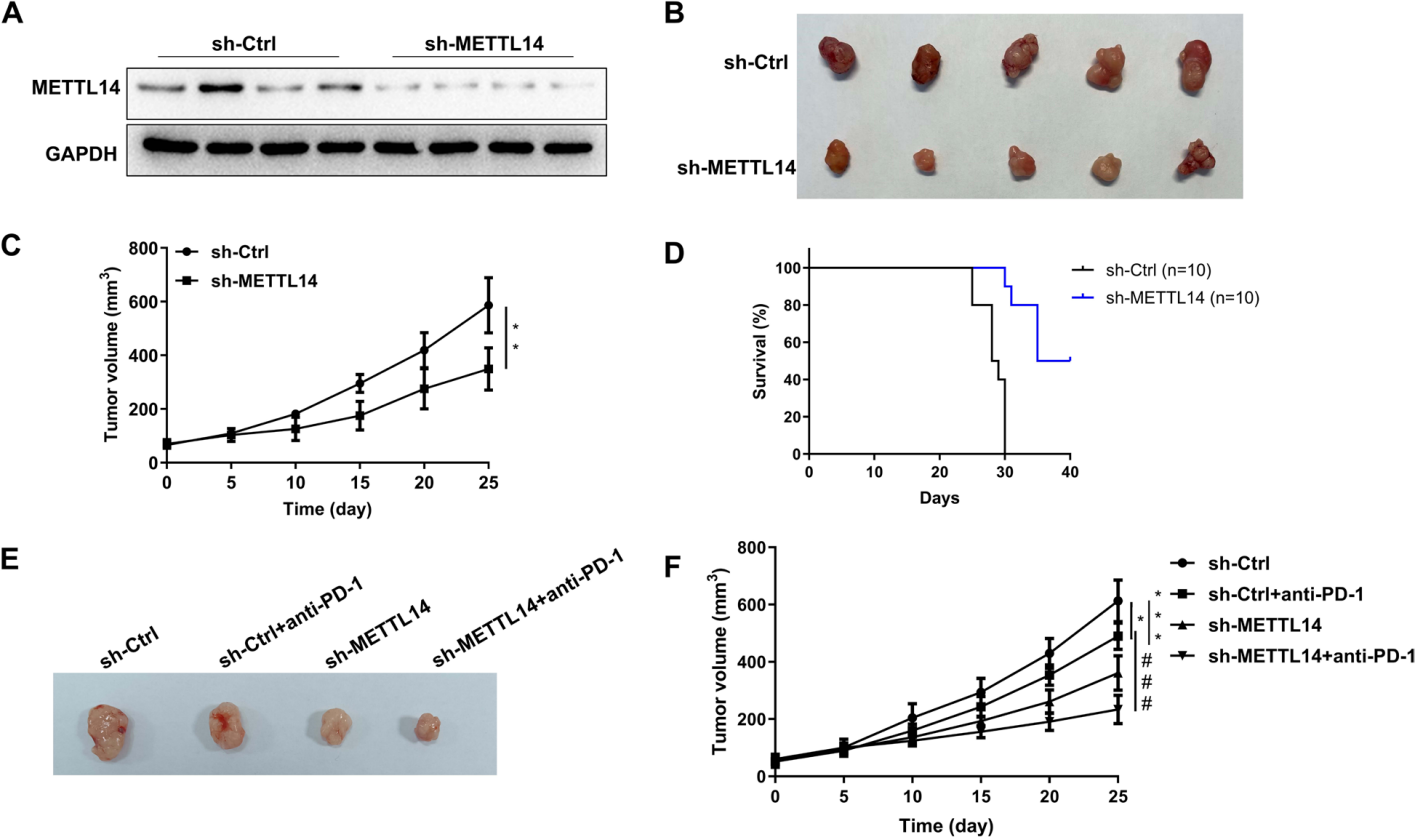
METTL14 knockdown inhibits tumor growth and enhances the response to PD-1 immunotherapy. After LLC cell injection (sh-Ctrl and sh-METTL14), lung cancer tissues were collected for further study. **A** Western blotting was used to assess METTL14 expression in mouse lung cancer tissues. **B** After modeling, the grafts were removed and photographed. **C** The volume of the transplanted tumor was measured every 5 days starting from day 5 to day 25. **D** 40-day survival in tumor-transplanted mice. ^**^P < 0.01 vs. sh-Ctrl. BALB/c mice in the sh-Ctrl and sh-METTL14 groups were injected with 10 mg/kg anti-PD-1at days 11, 14, 17, 20 and 23 after subcutaneous tumor transplantation, and the mice were killed on day 25. **E** After modeling, the grafts were removed and photographed. **F** The volume of the transplanted tumor was measured every 5 days from day 5 to day 25. ^*^P < 0.05, ***P < 0.001 vs. sh-Ctrl; ###*P* < 0.001 vs. sh-Ctrl + anti-PD-1

The effect of METTL14 knockdown was further verified in a syngeneic mouse model, and its effect on PD-1 treatment was studied. Western blot analysis revealed that knockdown of METTL14 decreased METTL14 protein levels in tumor tissues (Fig. 3A). The tumor volume was recorded every 5 days during modeling. After 25 days of modeling, the tumor volume in the sh-METTL14 group was significantly smaller than that in the sh-Ctrl group (*P* < 0.01, Fig. 3B-C). In addition, the 40-day survival rate of the mice treated with sh-METTL14 was significantly greater than that of the mice in the sh-Ctrl group (Fig. 3D). Subsequently, a syngeneic mouse model was treated with sh-METTL14 and an anti-PD-1 antibody to observe the effect of METTL14 on immunotherapy efficacy. At 25 days after surgery, the tumor volume in the anti-PD-1 antibody treatment group was significantly reduced compared with that in the sh-Ctrl group, and the effect in the sh-METTL14 treatment group was similar to that in the anti-PD-1 group (*P* < 0.05, *P* < 0.01; Fig. 3E-F). The tumor volume was significantly lower in the sh-METTL14 plus anti-PD-1 group than in the anti-PD-1 alone group (*P* < 0.001, Fig. 3E-F). These results demonstrated that METTL14 knockdown effectively enhanced the inhibitory effect of PD-1 treatment on transplanted tumors.

### METTL14 knockdown enhances CD8^+^T-cell activation and infiltration in tumor tissues

**Fig. 4:**
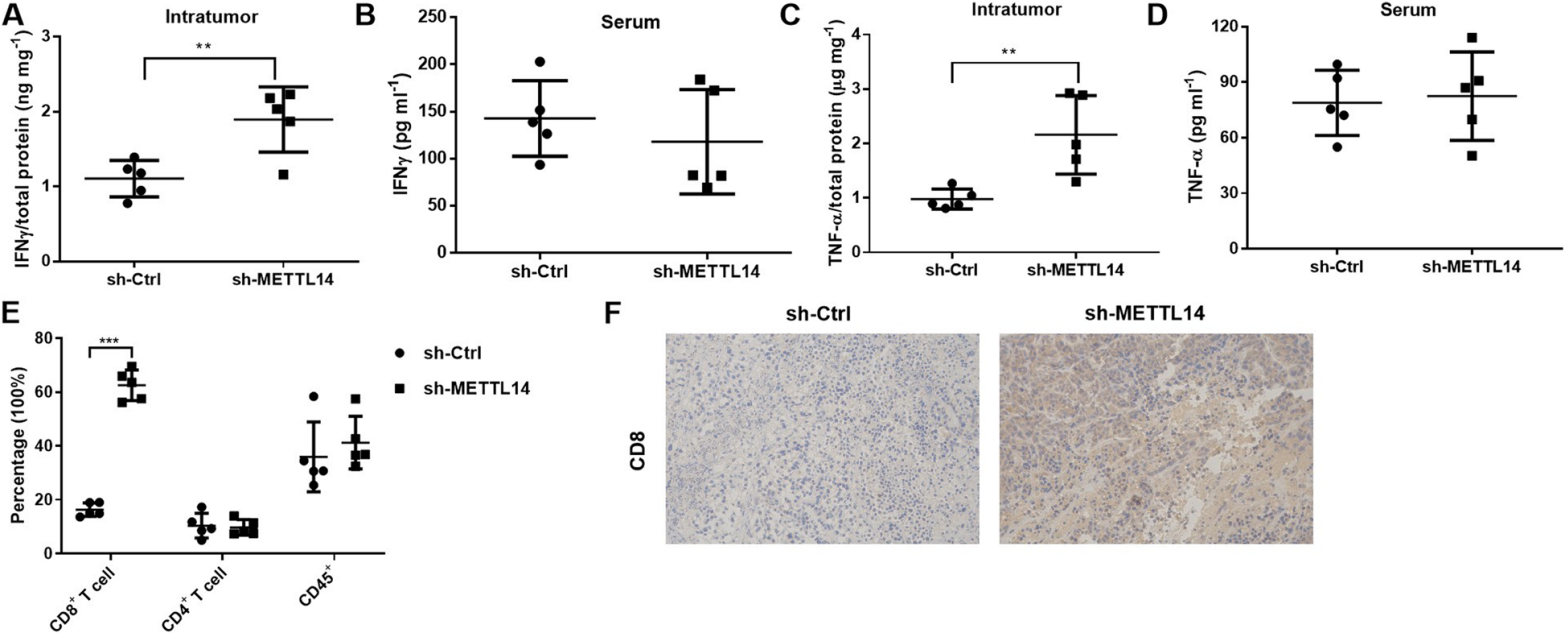
METTL14 knockdown enhances CD8^+^ T-cell activation and infiltration in tumor tissues. After LLC cell injection (sh-Ctrl and sh-METTL14), lung cancer tissues and serum samples were collected for further study. **A** Intratumor IFN-γ levels in thesh-Ctrl and sh-METTL14 groups were detected by ELISA. **B** Serum IFN-γ levels in thesh-Ctrl and sh-METTL14 groups were detected by ELISA. **C** Intratumor TNF-α levels in thesh-Ctrl and sh-METTL14 groups were detected by ELISA. **D** Serum TNF-α levels in thesh-Ctrl and sh-METTL14 groups were detected by ELISA. **E** The infiltrating CD8^+^ T cells, CD4^+^ T cells and CD45^+^ cells were detected by flow cytometry. **F** IHC staining was used to assess CD8-positive cells in lung cancer tissues. ^**^P < 0.01, ^***^P < 0.001 vs. sh-Ctrl

Next, the cellular mechanism by which METTL14 knockdown enhances the therapeutic effect of PD-1 was investigated. ELISA revealed that METTL14 inhibition significantly increased the intratumor IFN-γ concentration(*P* < 0.01, Fig. 4A). However, the serum IFN-γ level did not change between the sh-Ctrl group and the sh-METTL14 group (*P* > 0.05, Fig. 4B). Similarly, the intratumor TNF-α level was increased by METTL14 inhibition (*P* < 0.01, Fig. 4C). However, the serum TNF-α level did not change between the sh-Ctrl group and the sh-METTL14 group (*P* > 0.05, Fig. 4D). These changes in intracellular cytokines may be due to changes in the infiltrating components of immune cells. Therefore, the infiltrating fraction of immune cells in the transplanted tumors was subsequently analyzed by flow cytometry. In sh-METTL14-treated mice, the level of intratumoral infiltration of CD8^+^ T cells was significantly increased (*P* < 0.001, Fig. 4E), but the levels of CD4^+^ T cells and CD45^+^ cells were not significantly changed (*P* > 0.05, Fig. 4E). IHC staining revealed that the number of CD8 protein-positive cells was increased in the sh-METTL14-treated group (Fig. 4F). These results suggested that reducing METTL14 levels increased CD8^+^ T-cell infiltration.

### METTL14 knockdown increases stability by reducing the m6A methylation of HSD17B6 mRNA

**Fig. 5:**
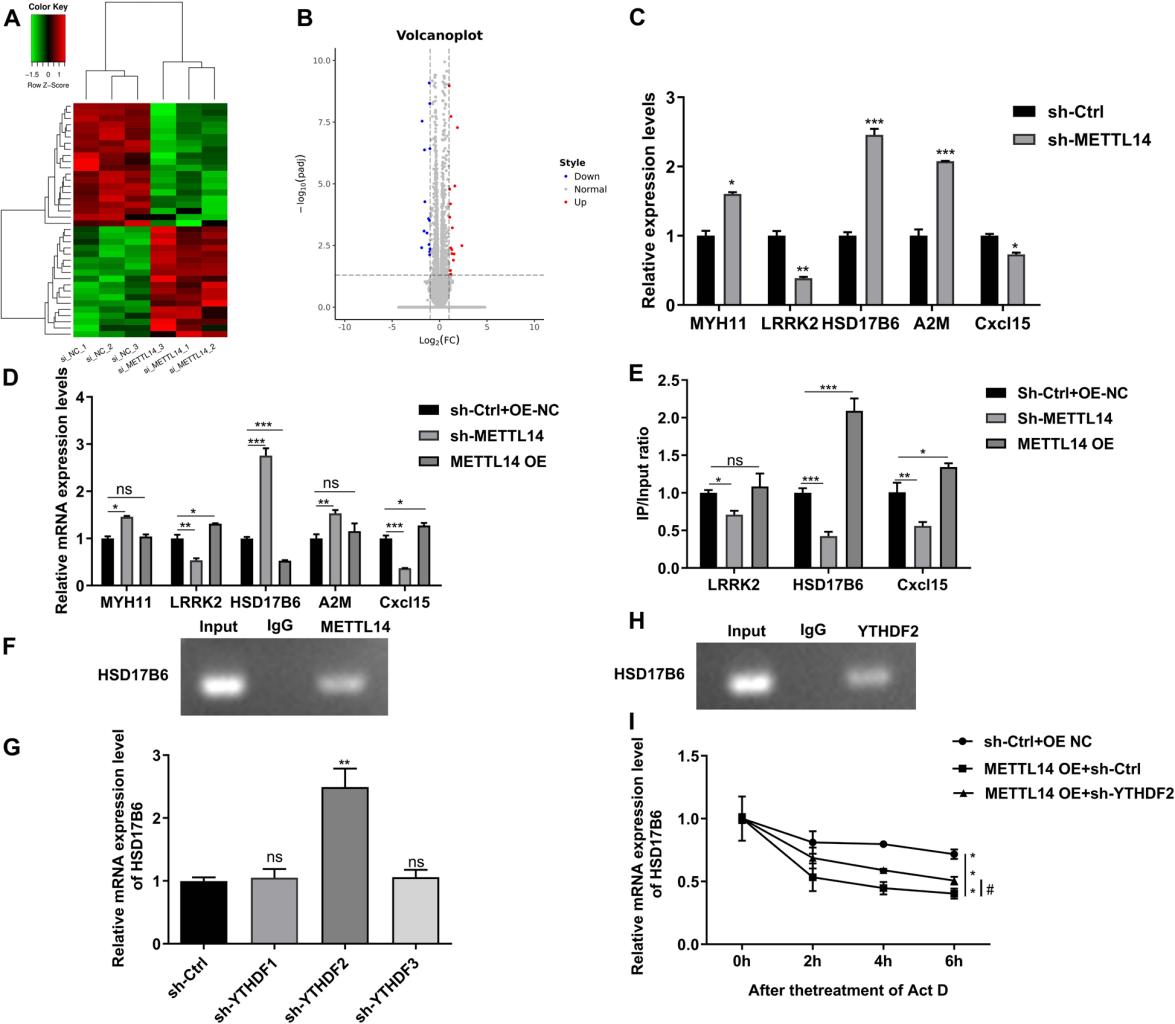
METTL14 knockdown increases stability by reducing the m6A methylation of HSD17B6 mRNA. After LLC cell injection (sh-Ctrl and sh-METTL14) was induced, lung cancer tissues were subjected to sequencing. **A** The differentially expressed genes were drawn as a heatmap. **B** The differentially expressed genes were drawn as a volcano map. **C** The top five DEGs with the most significant changes were selected for RT‒qPCR in mice injected with LLC cells (sh-Ctrl and sh-METTL14). ^*^P < 0.05, ^**^P < 0.01, ^***^P < 0.001 vs. sh-Ctrl. **D** RT‒qPCR was used to detect the differential expression of genes in LLC cells after sh-METTL14 and METTL14 overexpression transfection. **E** The level of m6A in the gene was detected by a MeRIP kit. *P < 0.05, **P < 0.01, ***P < 0.001 vs. sh-Ctrl + OE-NC. **F** RIP was used to detect the binding relationship between METTL14 and HSD17B6. **G** After the knockdown of YTHDF1-3 in LLC cells, the mRNA level of HSD17B6 was detected by RT‒qPCR. ^**^P < 0.01 vs. sh-Ctrl. **H** RIP was used to detect the binding relationship between HSD17B6 and YTHDF2. **I** The mRNA half-life of HSD17B6 was detected in LLC cells after sh-YTHDF2 and METTL14 overexpression transfection and actinomycin D treatment. ^***^P < 0.001 vs.sh-Ctrl + OE NC; # *P* < 0.05 vs METTL14 OE + sh-Ctrl

After syngeneic mice were subjected to sh-METTL14 treatment, the transplanted tumors were sequenced to analyze the DEGs. The expression of differentially expressed genes was plotted as heatmaps (Fig. 5A) and volcano plots(Fig. 5B). The five genes with the most differential expression between the sh-METTL14 group and the sh-Ctrl group were selected for RT‒qPCR. Compared to those in the sh-Ctrl group, the levels of MYH11, HSD17B6, and A2M were significantly upregulated in the sh-METTL14 group, while the levels of LRRK2 and Cxcl15 were downregulated in the sh-METTL14 group (*P* < 0.05, *P* < 0.01, *P* < 0.001, Fig. 5C). To further explore the effect of METTL14 on DEGs, the expression levels of the above five DEGs were detected after METTL14 knockdown and overexpression in cells; HSD17B6 was significantly upregulated after METTL14 knockdown (*P* < 0.001, Fig. 5D), and METTL14 overexpression suppressed HSD17B6 expression (*P* < 0.001, Fig. 5D). The changes in LRRK2 and Cxcl15 were opposite to those in HSD17B6, but MYH11 and A2M did not change significantly after METTL14 overexpression (Fig. 5D). After that, three genes (HSD17B6, LRRK2 and Cxcl15) whose expression significantly changed were selected for detection of m6A methylation levels via the MeRIP method, and HSD17B6 was found to be the most sensitive to changes in METTL14 levels(*P* < 0.001, Fig. 5E). RIP assays further revealed the binding relationship between METTL14 and HSD17B6 mRNA (Fig. 5F). Furthermore, the knockdown of YTHDF2 significantly altered HSD17B6 mRNA levels (Fig. 5G). Next, the binding relationship between YTHDF2 and HSD17B6 mRNA was also verified by the RIP method (Fig. 5H). The mRNA half-life of HSD17B6 was determined after actinomycin D treatment of LLC cells. METTL14 overexpression significantly accelerated the degradation of HSD17B6(*P* < 0.001, Fig. 5I), while the knockdown of YTHDF2 inhibited the degradation of METTL14 (*P* < 0.05, Fig. 5I).

### HSD17B6 overexpression promotes the activation of CD8^+^ T cells

**Fig. 6:**
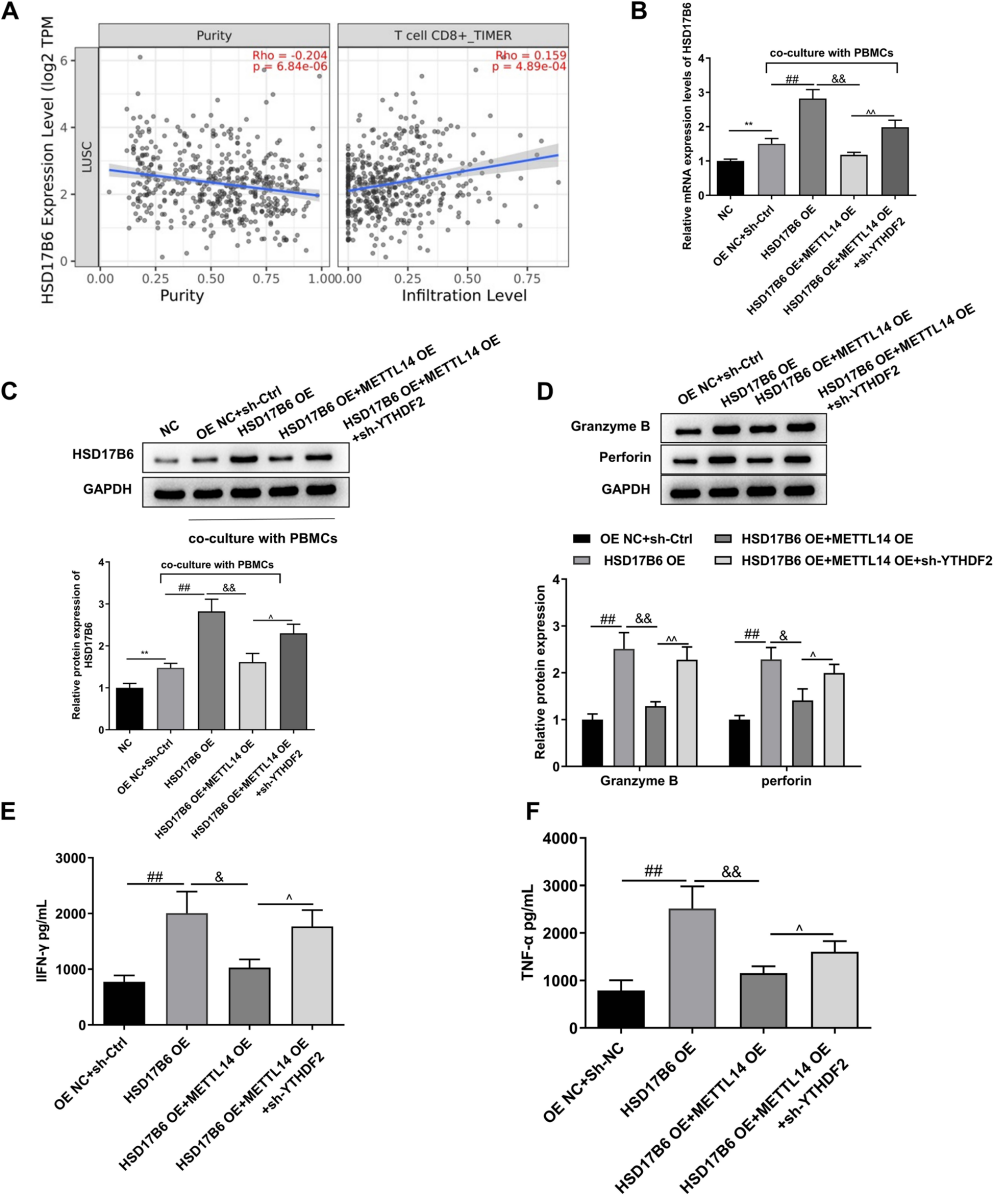
HSD17B6 overexpression promotes the activation of CD8^+^ T cells. **A** The online database TIMER (http://timer.cistrome.org/) was used to predict tumor HSD17B6 purity and correlation with CD8^+^T-cell infiltration. **B** RT‒qPCR was used to measure HSD17B6 levels in LLC cells treated with mouse PBMCs. **C** Western blotting was performed to detect HSD17B6 levels in LLC cells treated with mouse PBMCs. **D** The levels of the CD8^+^ T-cell effector Granzyme B and perforin-activated PBMCs in the coculture system were detected by western blot. **E** The level of IFN-γ in the PBMC supernatant was detected by an ELISA kit. **F** The level of TNF-α in the PBMC supernatant was detected by an ELISA kit.^**^P < 0.01 vs. NC; #*P* < 0.05, ##*P* < 0.01 vs.sh-Ctrl + OE NC; &&*P* < 0.01vs. HSD17B6 OE; ^*P* < 0.05, ^^*P* < 0.01 vs. HSD17B6 OE + METTL14 OE

The online database TIMER (http://timer.cistrome.org/) was used to predict the purity of HSD17B6 in tumors and the correlation of HSD17B6 with CD8^+^T-cell infiltration. HSD17B6 was negatively correlated with lung cancer tumor purity and positively correlated with CD8^+^ T cells (Fig. 6A). Next, LLC cells and activated mouse PBMCs were cocultured in vitro to mimic immune cell infiltration in the tumor microenvironment. RT‒qPCR revealed that the HSD17B6 level in LLC cells was significantly increased after coculture(*P* < 0.01, Fig. 6B). In the coculture system, HSD17B6 overexpression in LLC cells significantly increased the HSD17B6 mRNA level (*P* < 0.01, Fig. 6B), while METTL14 overexpression significantly inhibited the HSD17B6 overexpression-induced increase in the HSD17B6 mRNA level (*P* < 0.001, Fig. 6B). The knockdown of YTHDF2 significantly increased the level of HSD17B6 (*P* < 0.01, Fig. 6B). The HSD17B6 protein and mRNA levels showed similar trends(Fig. 6C). The levels of the CD8^+^ T-cell effector Granzyme B and perforin-activated PBMCs in the coculture system were detected by western blotting. The changes in these two effectors were also similar to the changes in HSD17B6 expression (Fig. 6D). Finally, the levels of IFN-γ and TNF-α in the PBMC supernatant were detected by ELISA. Consistently, the trends of IFN-γ and TNF-α were the same as those of HSD17B6 (Fig. 6E-F).

## Discussion

Lung cancer is a malignant tumor with high morbidity and mortality worldwide [1]. The occurrence and development of lung cancer is a multistage process involving multiple genes and pathway changes. Recent studies have reported that m6A modification regulates lung cancer development and tumor immunity and affects the prognosis of patients with lung cancer, which provides a potential therapeutic direction for lung cancer treatment ( [20, 21].

METTL14 is a m6A methylase that plays an important role in m6A methylation and has been found to be abnormally expressed in a variety of tumors [22–24]. For example, METTL14 is highly expressed in pancreatic cancer tissues, and the upregulation of METTL14 decreases PERP levels via m6A modification, promoting the growth and metastasis of pancreatic cancer [23]. In addition, METTL14 is significantly downregulated in colorectal cancer and inhibits the malignant process of colorectal cancer through the SOX4-mediated EMT process and PI3K/Akt signaling [24]. In the present study, we found that high METTL14 expression indicated a poor prognosis in patients with lung cancer. METTL14 has also been found to play a role in tumorigenesis [25]. For instance, Yang F et al. suggested that METTL14 knockdown inhibited non-small cell lung cancer malignancy by suppressing Twist-mediated activation of AKT signaling [25]. In the present study, METTL14 knockdown inhibited LLC cell growth, migration and invasion. Furthermore, in vivo assays revealed that METTL14 knockdown suppressed lung cancer progression.

METTL14 is closely associated with antitumor immunity [26, 27]. For instance, METTL14 regulates the immune response to anti-PD-1 treatment in colorectal cancer [26]. In addition, Wang X et al. reported that the downregulation of METTL14 activated antitumor immunity to participate in the delicaflavone-mediated inhibition of lung cancer [27]. In the present study, we found that METTL14 knockdown effectively enhanced the inhibitory effect of PD-1 treatment on transplanted tumors. Donnem T et al. reported that the number of CD8^+^T cells in the lung cancer microenvironment has a significant impact on the prognosis of lung cancer patients [28].This study suggested that CD8^+^T cells can be used as an independent prognostic factor in the lung cancer microenvironment. CD8^+^T cells secrete cytokines, including IFN-γ and TNF-α. Our study suggested that METTL14 knockdown significantly increased intratumor IFN-γ and TNF-α levels.Furthermore, we found that reducing METTL14 levelsincreased CD8^+^ T-cell infiltration.

m6A modification is regulated mainly by the recognition of m6A modification sites by reader proteins [29]. Reader proteins are YTH domain family proteins that mainly include YTHDF1, YTHDF2, and YTHDF3 [30]. YTHDF1 regulates the translation of mRNAs, whereas YTHDF2 increases mRNA degradation by reducing the stability of target transcripts, and YTHDF3 regulates the translation and degradation of mRNAs via specific biological processes [30]. For instance, Chen X et al. reported that METTL14 epigenetically elevates SOX4 expression via a m6A-YTHDF2-dependent pathway [24]. To further explore the role of METTL14, RNA-Seq, MeRIP and RIP were used to identify the downstream targets of METTL14. We found that HSD17B6 was negatively modulated by METTL14 and modified by METTL14-mediated m6A methylation. In the present study, we found that YTHDF2 knockdown increased HSD17B6 levels in LLC cells. Furthermore, the degradation of HSD17B6 was triggered by METTL14 overexpression, and knockdown of the m6A reader protein YTHDF2 suppressed HSD17B6 mRNA degradation. These data indicated that METTL14 knockdown induced HSD17B6 mRNA stability via a m6A-YTHDF2-dependent pathway.

Hydroxysteroid 17-beta dehydrogenase 6 (HSD17B6) is a vital protein in the synthesis of dihydrotestosterone [31]. The abnormal expression of HSD17B6 is closely associated with the progression of multiple tumors and can be used to assess the level of immune cell infiltration in tumor tissues [32]. HSD17B6 exhibited a strong association with tumor-infiltrating B cells, CD4^+^ and CD8^+^ T cells, neutrophils, dendritic cells, and macrophages [33].It has been proposed by earlier research that HSD17B6 may prevent tumor growth [31, 32]. For instance, HSD17B6 prevents liver cancer cell growth, migration, and invasion by regulating the expression of TGFB1 [32]. In addition, Tian T et al. showed that HSD17B6 was an independent potential prognostic biomarker for lung adenocarcinoma [31]. In addition, HSD17B6 suppressed lung adenocarcinoma progression by activating the Akt signaling pathway [31]. Here, we found that HSD17B6 was negatively correlated with lung cancer tumor purity and positively correlated with CD8^+^ T cells. In addition, HSD17B6 overexpression promoted the activation of CD8^+^ T cells.

In conclusion, our study explored the role of METTL14-mediated m6A modification in lung cancer progression and clarified the m6A-dependent regulatory mechanism involved. These findings revealed that METTL14 knockdown contributed to CD8^+^T-cell activation and the immunotherapy response to PD-1 via m6A modification of HSD17B6, thereby suppressing lung cancer progression.

### Supplementary Information

Supplementary Material 1.

## Data Availability

No datasets were generated or analysed during the current study.
